# Gas-containing mesenteric desmoid-type fibromatosis: A case report

**DOI:** 10.1097/MD.0000000000030326

**Published:** 2022-09-09

**Authors:** Tianjing Chang, Tang Sa, Mingchuan Yu, Bin Zhang, Zhe Lyu

**Affiliations:** a Department of Radiology, Shougang Hospital, Peking University, Beijing, China; b Pathology Department, Shougang Hospital, Peking University, Beijing, China.

**Keywords:** case report, computed tomography, desmoid-type fibromatosis, small mesenteric, surgery

## Abstract

Desmoid-type fibromatosis is a rare benign mesenchymal neoplasm. Only 8% of desmoid-type fibromatosis develops in the abdominal cavity. The mesentery is seldom affected and gastrointestinal stromal tumors need to be considered in the differential diagnosis, particularly when imaging examination shows a tumor containing gases in the abdominal cavity. Only a few cases of gas-containing mesenteric desmoid-type fibromatosis have been reported in the literature.

**Patient concerns:**

A 69-year-old male patient presented with hematochezia and intermittent upper abdominal pain.

**Diagnosis:**

Contrast-enhanced computed tomography revealed a 3.9 × 3.6 cm gas-containing mass infiltrating the third portion of the duodenum. The tumor was heterogeneous, with cysts and air bubbles. It showed heterogeneous weak-to-mild enhancement in the solid part. Postoperative pathological examination confirmed a final diagnosis of mesenteric desmoid-type fibromatosis.

**Interventions:**

The patient underwent surgical resection of intra-abdominal lesion.

**Outcomes:**

No evidence of local recurrence was noted during the 6 months of follow-up.

**Lessons:**

Accurate preoperative diagnosis is difficult for an intra-abdominal gas-containing mass on computed tomography scan. The appearance of spiculated infiltrative margin suggests the diagnosis of desmoid-type fibromatosis. Further investigation of imaging evidence and treatment methods is necessary.

## 1. Introduction

Desmoid-type fibromatosis (DF), also known as desmoid tumor or deep fibromatosis, is rare (0.03% of all tumors)^[1]^ benign neoplasm that lacks the potential to metastasize. However, it is locally infiltrative and shows a strong tendency for local recurrence after surgical resection.^[2]^

DF can be classified as extra-abdominal, intra-abdominal, or abdominal walls, according to its location and only 8% of DF develop from the mesentery and retroperitoneum,^[3]^ where it resembles a gastrointestinal stromal tumor (GIST). The clinical course and treatment strategies for DF and GIST are different, and misdiagnosis may lead to inappropriate therapeutic decisions. Owing to the stabilization and regression capacity of DF, an active surveillance period is recommended as an initial treatment strategy.^[4]^ Computed tomography (CT) is a reliable imaging exam to evaluate the involvement of adjacent organs, and the air in the tumor is considered an indication of its intestinal origin. This paper reports a rare case of gas-containing mesenteric DF misdiagnosed as a GIST preoperatively. Ethical approval was obtained and the patient provided informed consent.

## 2. Case presentation

**Figure 1.**
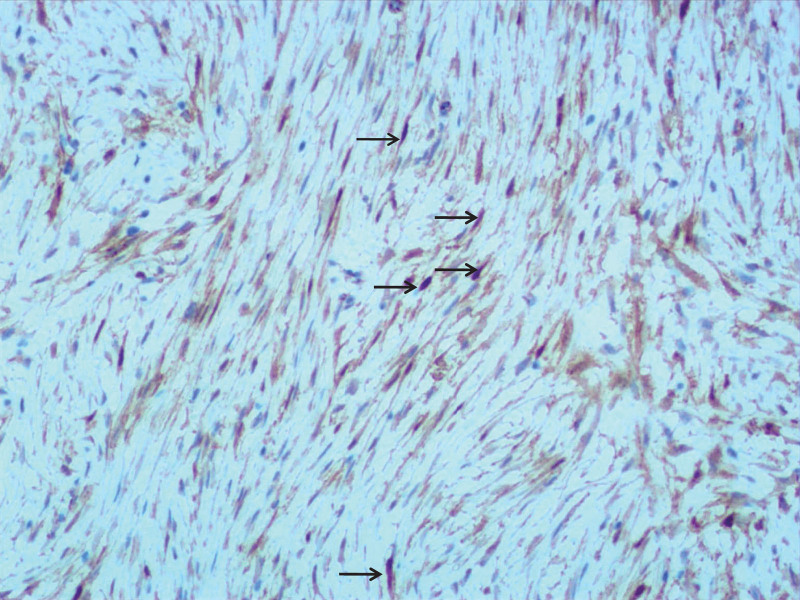
Arterial phase enhanced CT images of the lesion. (A) Axial CT images showing an air-containing mass in the abdominal cavity(arrow). (B, C) Sagittal and coronal CT images showing that the lesion (arrow) was closely related to the third portion of duodenum. CT = computed tomography.

**Figure 2.**
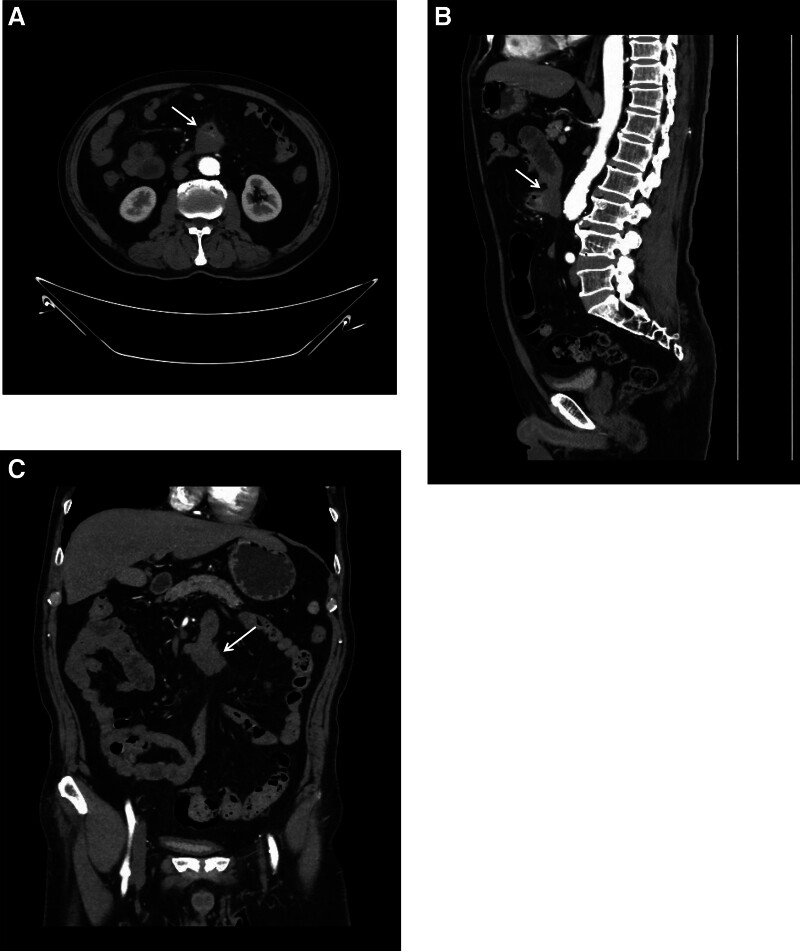
Specimen view in section.

**Figure 3.**
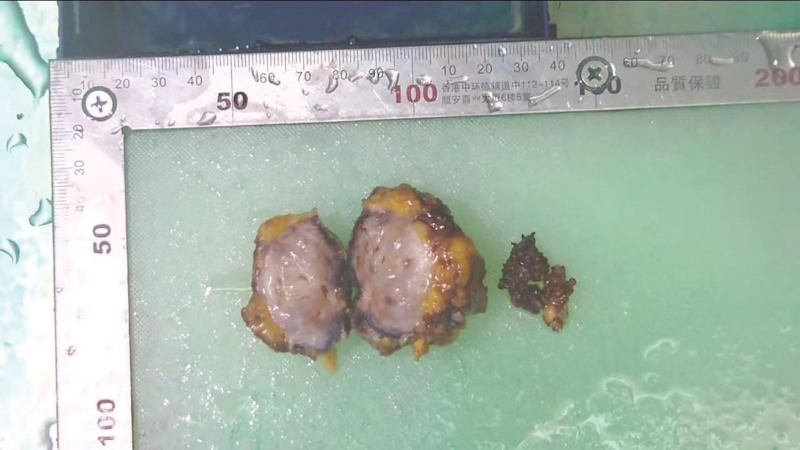
Immunohistochemical staining showing strong positive β-catenin (arrow).

A 69-year-old man was admitted to the Department of Gastrointestinal Surgery due to hematochezia and intermittent upper abdominal pain for more than a year. The patient presented with intermittent blood in the stool and epigastric pain for 1 year. There was no abdominal distension or diarrhea. No emesis or palpable mass. His abdomen was soft and had epigastric tenderness. No rebound tenderness or obvious masses were touched. Laboratory evaluation results were unremarkable. The contrast-enhanced CT of the abdomen and pelvis revealed a 3.9 × 3.6 cm gas-containing mass infiltrating the third portion of the duodenum. The tumor was heterogeneous with cysts and air bubbles. It showed heterogeneous weak-to-mild enhancement in the solid part with nonenhancing cystic areas. The lesion had spiculated infiltrative margins (Fig. 1). The patient had a history of hypertension for 20 years and the maximum recorded blood pressure was 200/110 mm Hg. He also had a history of diabetes for 2 years. The patient underwent surgical resection of intra-abdominal lesion. The hard tumor was found at the root of the mesentery, and the lesion adhered closely to the third portion of the duodenum. The duodenum was invaded and the lesion was completely removed with part of the duodenal wall (Fig. 2). The inferior mesenteric artery was closely related to the mass; therefore, it was excised and ligated. Histological findings confirmed the diagnosis of spindle cell tumor infiltrating mesenteric adipose tissue and muscularis propria of the intestinal wall. Immunohistochemical examination showed CD117 (–); Dog-1 (–); CD34 (–); S-100 (–); SMA(–); Desmin (–); Ki-67 (2% +); SDHB (+); β-catenin (+); caldesmon (–); Vimentin (+); ALK (–) (Fig. 3). The final diagnosis was desmoid-type mesenteric fibromatosis. The patient was discharged 32 days postoperatively, without complications. No evidence of recurrence was noted for 6 months after surgery.

## 3. Discussion

Desmoid-type fibromatosis (DF) is a benign mesenchymal tumor that is considered locally aggressive without the capacity for metastases.^[5,6]^ The tendency for local recurrence after surgical resection and invasion of surrounding structures^[2]^ makes DF a significant cause of morbidity and mortality.

DF accounts for 3% of soft-tissue neoplasms, with an incidence of 2 to 4 cases per million per year according to reports. It typically affects individuals aged 15 to 60, with a peak incidence of 30 to 40 years.^[7,8]^ Although mostly sporadic, approximately 5% to 10% of cases are associated with hereditary syndromes such as familial adenomatous polyposis and Gardener syndrome.^[9]^

Patients have a varying pattern of clinical symptoms according to the tumor site, origin, and size.^[10]^ Most patients are asymptomatic. Some patients may experience abdominal pain, discomfort, vomiting, palpable masses, gastrointestinal bleeding, or symptoms caused by organ compression or invasion.^[11]^

The clinical course of DFs remains unpredictable. In this case, the patient had gastrointestinal bleeding and intermittent upper abdominal pain for 1 year without other symptoms.

Because DF and GIST differ widely in biological behavior, pathology, and prognosis, it is very important to correctly identify lesions.^[12]^ Imaging examination is essential not only for diagnosis but also for differences between the 2.

Mesenteric DF is generally seen as a soft-tissue lesion with smooth or infiltrative margins^[13]^ and patients without familial adenomatous polyposis tend to have smooth margins.^[14]^

Typical CT findings of DF are well-circumscribed, homogeneous, and solid tumors with weak enhancement. They have variable attenuation because of the relative abundance of collagens or myxoid stroma.^[15]^ However, most cases present as homogenous lesions because necrotic changes and hemorrhage are rare.^[16]^

The DF may have spiculated infiltrative margins and the edge may be indistinct due to the infiltration of adjacent structures.

Primary GISTs are typically well-defined masses with heterogeneous enhancement caused by necrosis and hemorrhage.^[17]^ The gas is relatively common in the GIST, which indicates necrotic areas and the presence of a fistula in the gastrointestinal lumen.^[4]^ In this case, the presence of air within the lesion led to a misdiagnosis of a GIST.

**Figure 4.**
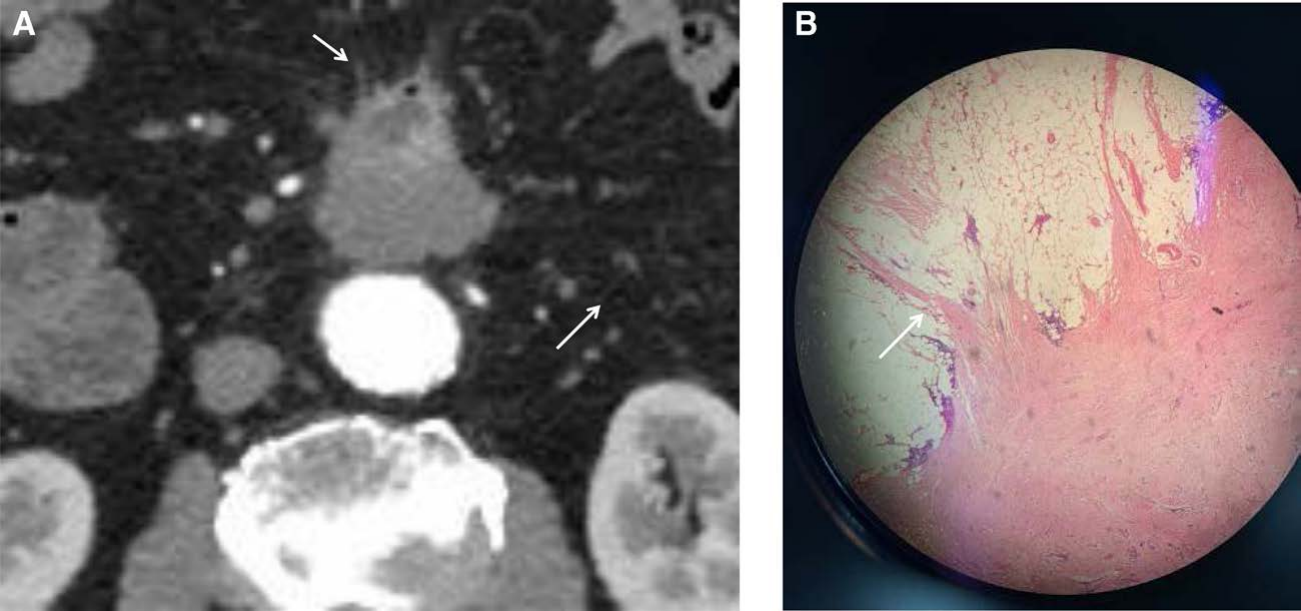
The spiculated margin (arrow) of the lesion on CT and the infiltrative growth of tumor cells (arrow) observed under microscope. CT = computed tomography.

However, spiculated margins can be seen in this case, indicating the infiltrative growth of the lesion in the surrounding adipose tissue. It has been reported as an independent predictor of DF recurrence.^[18]^ The appearance of this sign does not look consistent with the typical characteristics of GIST. Under the microscope, the spiculated margin of the lesion contained tumor cells infiltrating the surrounding adipose tissue (Fig. 4). This indicates that spiculated margins can accurately reflect the infiltrative growth pattern.

On gross examination, mesenteric DF is usually a nonencapsulated, well-circumscribed lesion, but its margins may be irregular and penetrate microscopically into the viscera and peripheral tissues.^[14]^

Mesenteric GISTs should be factored into the differential diagnosis of mesenteric DF. GIST generally contains areas of hemorrhage and necrosis, whereas focal necrosis is not a typical feature of mesenteric DF.^[19]^

The histopathological characteristics of DF and GIST may sometimes be confused, but immunohistochemical staining contributes to differentiation.^[20]^ The β-catenin protein is believed to play a crucial role in the development of DF, and nuclear β-catenin positivity supports its diagnosis, which is absent in GIST. CD117 is consistently negative in DF and is commonly positive in GIST. SMA, Desmin, and S-100 have no effect on distinguishing.

Treatment of DF remains controversial. To optimize the treatment of DFs, a multidisciplinary approach is required for individual patients. Currently, an active surveillance period is recommended as an initial treatment strategy.^[4]^

Active treatments, including surgery, radiotherapy, and systemic therapy, are multiple.

Surgical resection was regarded as the principal approach by some scholars. Despite the treatment modality, the local recurrence rate for DF is 30% to 40%.^[21,22]^ Over 80% of recurrences occur within 3 years after treatment.^[23]^ Given the high recurrence rate, close follow-up is essential.

## 4. Conclusion

We described the diagnosis and surgical treatment of a case of gas-containing small intestinal mesenteric DF. Due to intestinal invasion, the accurate imaging diagnosis prior to surgery was difficult, but the appearance of spiculated margin suggested the diagnosis of DF. Currently, there are few cases of gas-containing DF and more imaging evidence and treatment methods should be investigated.

Written informed consent was obtained from the patient for publication of the case details and accompanying images.

## Author contributions

Resources: Tang Sa.

Writing—original draft: Tianjing Chang.

Writing—review & editing: Mingchuan Yu, Bin Zhang, Zhe Lyu.
